# Improving the Sexual Wellbeing of Patients with Psychotic Illness

**DOI:** 10.1192/j.eurpsy.2024.1536

**Published:** 2024-08-27

**Authors:** N. Stanton, E. Angova, K. Diamantopoulos

**Affiliations:** General Adult Psychiatry, Central & North West London NHS Foundation Trust, London, United Kingdom

## Abstract

**Introduction:**

Sexual dysfunction (SD) is common in psychotic illness including schizophrenia, occurring in 30-82% of patients. It negatively impacts wellbeing and antipsychotic compliance, resulting in higher risk of relapse and hospitalisation. Due to over-reliance on spontaneous reports from patients, SD is typically under-identified which prevents investigation and treatment.

**Objectives:**

To establish whether SD is under-identified in patients with psychosis in a general adult community mental health team; to elicit whether the Arizona Sexual Experience Scale (ASEX) improves identification; to investigate and manage identified cases of SD; to make recommendations about identification and monitoring of SD in this patient population.

**Methods:**

A 12-month retrospective audit of patients with psychosis prescribed a long-acting injectable (LAI) antipsychotic (n=36) to identify sexual symptoms was completed. The ASEX was subsequently issued to screen for SD.

**Results:**

Audit: 3/36 (8%) patients had documented sexual symptoms. Of the 18/36 patients that completed the ASEX: 10 (56%) exhibited SD. 4 consented to further investigation. 5 patients experienced significant difficulties with the language used in the ASEX. At the end of the project we revised the ASEX with simpler, colloquial language.

**Conclusions:**

Implementation of the ASEX results in clear improvements in identification and monitoring of SD. Maudsley Practice Guidelines can inform investigation and management of SD. We suggest a review of NICE guidance to incorporate the above into clinical practice. Further work is needed to establish whether the revised ASEX can be developed and validated.

**Disclosure of Interest:**

None Declared

